# Strategies for preventing school bullying—A life education perspective

**DOI:** 10.3389/fpsyg.2024.1429215

**Published:** 2024-09-18

**Authors:** Jason Cong Lin, Yi-Huang Shih

**Affiliations:** ^1^Department of International Education, The Education University of Hong Kong, Hong Kong, Tai Po, Hong Kong SAR, China; ^2^Center of Teacher Education, Minghsin University of Science and Technology, Hsinchu, Taiwan

**Keywords:** anxiety, depression, life education, loneliness, school bullying, stress

## 1 Introduction

The implementation of education affects a country's development and success, and education is a catalyst for personal development and a powerful tool for global change. Its influence extends beyond the development of skills required for economic success. It can contribute to nation-building and reconciliation. This affirms education's essential role in life (Wang and Shih, 2022; Muweesi et al., 2024).

Challenges such as school bullying often occur in educational activities. Bullying is a complex social relationship problem that typically involves repeated, deliberate intent to harm or intimidate others by exploiting power imbalances. Bullying is generally ongoing and measured in terms of frequency (e.g., daily) and duration (e.g., during the previous school term). Although extensive research has been conducted on the topic, bullying remains a global concern that can have long-term negative personal, social, emotional, academic, and economic consequences (Deborah et al., 2023; Green et al., 2023). Bullying is one of the most prevalent problems in schools. The term bullying encompasses physical and verbal aggression, prejudice, and discrimination. Parents, family members, and teachers have become increasingly concerned about this problem (Barros, 2024).

Most antibullying curricula in schools are based on a social–ecological perspective. Whole-school approaches to developing antibullying policy from this perspective have the potential to empower school communities to address aggression by involving parents, teachers, administrators, and community members. This opinion piece explored how life education can enhance a social–ecological approach to antibullying programs (Donoghue et al., 2023).

## 2 School bullying

Bullying is a pervasive problem that impacts children and youth in schools involved as perpetrators, victims, and bystanders. Bullying is a form of violence and often has psychological effects. The targets of bullying are typically individuals perceived to be weak or unpopular, and the aim of the action is to intimidate and disempower such individuals (Nickerson, 2019; Huang, 2023).

School bullying involves direct or indirect continual use of language, words, pictures, symbols, body movements, or other means by individuals or groups within or between schools, inside or outside the school setting. Bullying individuals or groups engage in behaviors that deliberately belittle, exclude, bully, harass, or tease others, which creates a hostile or unfriendly learning environment that is difficult to cope with and causes mental, physical, or property damage, thereby disrupting normal learning activities (Wu and Lin, 2005; Nickerson, 2019).

School bullying is a form of bullying that occurs within schools that is characterized by intentionally harmful and recurring behavior that targets the same children. Bullying has existed in human society for a considerable amount of time. Among the various forms of bullying, violent bullying of children has garnered particular attention. Bullying can also involve oppressive behavior resulting from unequal power dynamics among children. Bullying can be considered to have occurred any time a student experiences prolonged and repeated bullying or harassment by one or more students inside or outside the school that can cause physical and mental pain (Wu and Lin, 2005).

## 3 Life education

In fact, the meaning of life is love. That is, to be loved and to love. From another perspective, the meaning of life is to recognize the presence of love and expand one's life with love. Without love, life has no meaning. Love is the core of life. Life is the starting point and the primary focus of all educational activities. Creating conditions that support individual life development is a core focus of education. Education should foster healthy development of life force, and it encompasses various elements that have potential to boost vitality (Yao, 2023; Shih, 2024).

Life education guides students in transitioning from a focus on limited, short-term desires to a focus on unlimited, long-term intangible value. It helps them understand life's infinite possibilities, harness their life force, and achieve their life goals. Life education involves the meaning, ideals, and practice of being human and focuses on deepening one's outlook on life, internalizing values, and integrating mobility. Therefore, it helps students establish a complete outlook on life and personal values (Shih, 2022a; Yao, 2023).

The goal of life education is to help students discover meaning in life and realize their potential throughout their life journey. It teaches students to understand themselves, cherish life, respect others, care for all living things, live authentically, realize their potential, and contribute to the public good. In life education, students explore the entire process of life; they learn to face and solve problems with a positive and optimistic attitude, which can improve their quality of life (Sun, 2000; Shih, 2020a).

## 4 Discussion: strategies for preventing school bullying from a life education perspective

### 4.1 Implementation of life education to reduce students' loneliness to prevent school bullying

By exploring the meaning of life, life education teaches individuals to face and solve problems with a positive and optimistic attitude, thus improving their quality of life (Sun, 2000). Feelings of loneliness arise when people lose their sense of meaning in life and fail to understand their life's value. Implementing life education to reduce students' loneliness and prevent school bullying involves fostering a supportive and inclusive school environment (Sun, 2000). Life education can encompass various aspects, including emotional intelligence, social skills, empathy, and community building. Here are some strategies that can be implemented.

#### 4.1.1 Social-emotional learning programs

Integrating Social-emotional learning (SEL) into the curriculum to teach students skills like empathy, self-awareness, and emotional regulation. These skills can help students better understand their own emotions and those of others, reducing feelings of isolation and increasing positive social interactions (Jones and Bouffard, 2012; Kim et al., 2024).

#### 4.1.2 Peer support systems

To establish peer mentoring or buddy systems where older or more socially adept students support younger or more isolated peers. This can help foster connections and reduce feelings of loneliness among students (Seery et al., 2021; Hayman et al., 2022).

#### 4.1.3 Inclusive activities and clubs

Feelings of acceptance within school communities can promote positive psychological outcomes. Despite occurring outside of the classroom, youth who engage in extracurricular activities typically report greater school belonging. Teachers should encourage students participate in group activities, clubs, and extracurricular programs that emphasize teamwork, cooperation, and inclusion. These settings provide opportunities for students to form friendships and feel a sense of belonging (O'Donnell et al., 2023).

#### 4.1.4 Bullying prevention programs

Implementing comprehensive bullying prevention programs that educate students about the impact of bullying, promote positive behavior, and provide clear guidelines for addressing bullying incidents. Creating a safe environment where students feel supported can reduce the prevalence of bullying (Gaffney et al., 2021).

#### 4.1.5 Mindfulness and wellbeing practices

The use of mindfulness in schools has greatly expanded over the past 10 years. Research has demonstrated positive psychological effects of mindfulness for students as well as teachers. Teachers should incorporate mindfulness practices and discussions on mental health into the school routine. This can help students manage stress and emotions, leading to a more positive school experience (Garro et al., 2023).

#### 4.1.6 Self-awareness

Self-awareness is often seen as a critical component in leadership and career success. Self-awareness is the ability to recognize and understand one's own thoughts, emotions, and behaviors. It involves a conscious knowledge of one's character, feelings, motives, and desires. Here are some key points about self-awareness: (1) emotional self-awareness: understanding one's own emotions and their impact; (2) accurate self-assessment: recognizing one's strengths and limitations; (3) self-confidence: a sense of self-worth and capabilities (Axelrod, 2012; Carden et al., 2022).

#### 4.1.7 Parental and community involvement

Engage parents and the community in life education efforts. Workshops and seminars can educate parents on supporting their children's emotional and social development at home, creating a more cohesive support system (Shih, 2022a).

#### 4.1.8 Counseling and support services

Ensure access to counseling services for students who may need additional support. School counselors can provide a safe space for students to discuss their feelings and work through challenges.

#### 4.1.9 Curriculum integration

Integrate themes of empathy, respect, and community into various subjects. For example, literature and history classes can explore stories and events that highlight these values (Shih, 2022b).

#### 4.1.10 Teacher training

Provide teachers with training on identifying signs of loneliness and bullying and strategies for fostering a supportive classroom environment.

By focusing on these areas, schools can create a nurturing environment that promotes positive relationships, reduces loneliness, and ultimately prevent school bullying.

### 4.2 Implementation of life education to reduce students' stress, depression, and anxiety levels and prevent school bullying

School bullying is a critical problem of global concern and potentially leads to serious health consequences for students. Research indicates that bullying is a significant risk factor for adolescent mental and physical health in the short and long term (Wang and Chen, 2024). The prevalence of bullying among students in schools has increased rapidly. Bullying can lead to various problems such as stress, depression, anxiety, and impaired academic performance (Yosep et al., 2024). Research indicates that significant correlations exist between school bullying experiences and psychological problems (Zhao et al., 2024).

Life education is an integrative, experiential, and continual form of whole-person education that places students at the center of teaching design, with various teaching activities used to achieve goals related to knowledge, emotion, and intention. The main axis of the Life Education Curriculum is the connotation of life education, and units within the curriculum, such as “Knowing Yourself,” “Knowing Others,” “Emotional Education,” and “Care and Cooperation,” are designed in a step-by-step manner. The teaching design and arrangement of the curriculum enables students to relieve stress, feelings of depression, and anxiety while enhancing their emotional development (Sun, 2000; Shih, 2022a).

In conclusion, implementing life education in schools can be an effective way to address and reduce students' stress, depression, anxiety, and school bullying. Here are some strategies that can be employed.

#### 4.2.1 Curriculum integration

##### 4.2.1.1 Emotional intelligence

Young people go through extreme ups and downs at different stages of their lives, especially during puberty. Without proper support and guidance, some children and adolescents can find it hard to understand why they have unhelpful responses to their emotional reactions. Teachers should incorporate lessons that teach students about emotional awareness, regulation, and empathy. This can help them understand their own emotions and those of others, leading to more positive interactions (Quan and Yao, 2014; Gonzales, 2022).

##### 4.2.1.2 Mindfulness and stress management

Teach techniques such as mindfulness, meditation, and relaxation exercises to help students manage stress and anxiety (Garro et al., 2023).

##### 4.2.1.3 Resilience and coping skills

Develop students' resilience by teaching coping strategies for dealing with setbacks, failures, and challenges.

##### 4.2.1.4 Emotional engagement

Emotional engagement is important for behavioral and psychological outcomes has also been explored in the school connection literature. Emotional engagement refers to the extent to which individuals are involved, invested, and emotionally connected to an activity, task, or environment. In educational settings, emotional engagement is crucial for fostering a deep connection to learning and enhancing overall academic experience. For students, emotional engagement can be fostered through interactive learning. Hands-on activities and interactive lessons help students feel more involved and interested. In addition, supportive and nurturing relationships with teachers and peers enhance emotional investment (Markowitz, 2017; Luo et al., 2019).

#### 4.2.2 Supportive environment

##### 4.2.2.1 Emotional safety

Emotional safety in schools refers to how safe a student feels in expressing their emotions in school. Students should feel secure and confident as they express themselves and take on challenges that encourage them to try something new. Emotional safety is considered a defining component of a positive learning environment and is related to psychological wellbeing, and positive academic and social outcomes. In schools, emotional safety is developed through supportive relationships. Teachers should create a school environment where students feel safe to express their feelings and concerns, and develop students' sense of emotional safety. This can include counseling services, peer support groups, and designated areas where students can take a break if they feel overwhelmed (Shean and Mander, 2020; National Center on Safe Supportive Learning Environments, 2024).

##### 4.2.2.2 Anti-bullying policies

Implementing clear policies and procedures for addressing bullying, including prevention programs, reporting mechanisms, and consequences for bullying behavior.

#### 4.2.3 Teacher training and support

##### 4.2.3.1 Professional development

Schoolteachers are often the first to respond when a student presents with a mental health issue in the classroom. Therefore, provide teachers with training on recognizing signs of stress, anxiety, and depression in students. Equip them with skills to provide appropriate support or referrals to professional help (Gunawardena et al., 2024).

##### 4.2.3.2 Modeling positive behavior

Encourage teachers to model positive behaviors, such as active listening, empathy, and respectful communication, to create a supportive classroom atmosphere.

#### 4.2.4 Regular assessment and feedback

##### 4.2.4.1 Monitoring mental health

Regularly assess students' mental health and wellbeing through surveys, interviews, or observation. Use this data to inform and adjust programs as needed (Park et al., 2020).

##### 4.2.4.2 Student feedback

Encourage students to provide feedback on the life education programs, ensuring they are meeting the students' needs and preferences.

#### 4.2.5 Promotion of positive peer relationships

##### 4.2.5.1 Social skills training

Social skills training is one of the oldest and widely studied approaches to psychiatric rehabilitation. Teach social skills for students, such as effective communication, conflict resolution, and teamwork, to help students build positive relationships with their peers (Mueser and Bellack, 2007).

##### 4.2.5.2 Peer mentorship programs

Establish peer mentorship or buddy systems to support students who may be struggling with social integration or emotional challenges (Seery et al., 2021; Hayman et al., 2022).

#### 4.2.6 Incorporating cultural sensitivity

##### 4.2.6.1 Cultural awareness

Include components of cultural awareness and sensitivity in the curriculum to foster understanding and respect among students from diverse backgrounds (Shih, 2020b).

##### 4.2.6.2 Tailored programs

Adapt life education programs to reflect the cultural and social context of the students, making them more relevant and effective.

By implementing these strategies, schools can create a more supportive and inclusive environment that promotes students' overall wellbeing and reduces the incidence of stress, depression, anxiety, and bullying.

## 5 Reflections and conclusions

### 5.1 Reflections

Bullying occurs when students repeatedly subject a peer to negative activities. It is a problem involving unsocial and rule-breaking behavior. Bullies are often impulsive and have a strong need to dominate others; they lack empathy and are generally physically stronger than their victims. Conversely, victims tend to be vigilant, sensitive, and quiet students with low self-confidence. They often lack social competencies that could help them divert bullying, such as the ability to use humor. When bullied, they typically respond emotionally, shedding tears or becoming irritated, which often encourages the bully. Childhood bullying has increasingly been reported to be one of the most common and widespread forms of school violence (Taj et al., 2024).

School bullying can occur in the neighborhoods surrounding schools in addition to in the schools themselves. Traditionally, governance of school bullying has focused on visible and physical harm to victims and has not accounted for the psychological problems caused by bullying behavior, which can pose a danger to the lives of those being bullied. Consequently, school bullying remains a major problem that affects students' academic achievement and general wellbeing worldwide. It is a complex phenomenon that varies across cultural and regional contexts, and understanding the nuances of these contexts is crucial to the development of effective interventions (Hasibuan and Rizana, 2023; Jin, 2023).

### 5.2 Conclusions

School bullying is particularly prevalent among teenagers. Bullying behavior can have detrimental effects on victims and perpetrators, potentially leading to lower academic achievement, problems with socialization, and disruption of physical and mental health. Intervention is required to address the problem of bullying (Yuhbaba et al., 2023).

This opinion piece proposes that life education can help address the problem of school bullying and suggests the following prevention strategies: (1) implementing life education to reduce students' loneliness and prevent school bullying and (2) implementing life education to reduce students' stress, depression, and anxiety levels.

In conclusion, exposure to bullying at school is strongly associated with loneliness. Implementing life education can reduce bullying at school and enable development of effective interventions to mitigate the persistent loneliness associated with bullying among adolescents. Because bullying and loneliness frequently occur in schools, they are ideal settings for bullying-focused interventions. Interventions implemented in school settings can be used to target the entire adolescent population, and evidence supports the effectiveness of life education interventions in preventing school bullying (Madsen et al., 2024). Greater emphasis has been placed on the importance of life education in the literature. The current study may assist with the development of life education curricula in schools and with reducing students' emotional distress, anxiety, and bullying behaviors and therefore serves as a valuable contribution to the literature on the prevention and treatment of school bullying.
